# How can medical schools contribute to bringing about health equity?

**DOI:** 10.1186/2045-4015-3-17

**Published:** 2014-05-27

**Authors:** Mary CJ Rudolf, Shmuel Reis, Trevor J Gibbs, Deborah Murdoch Eaton, David Stone, Michael Grady, Anita Berlin, Mitch Blair, Jumanah Essa-Hadad, Sivan Spitzer-Shohat, Michael Weingarten

**Affiliations:** 1 Bar Ilan Faculty of Medicine in the Galilee, POB 1589, Henrietta Szold 8, 1311502 Safed, Israel; 2 Association for Medical Education in Europe, 5 Meols Court, 6 Meols Drive, Hoylake, CH47 4AQ Wirral, UK; 3 University of Sheffield, Beech Hill Road, S10 2RX Sheffield, UK; 4 University of Glasgow, School of Medicine, Wolfson Medical School Building, University Avenue, G12 8QQ Glasgow, UK; 5 Institute of Health Equity, University College London, 1-19 Torrington Place, WC1E 7HB London, UK; 6 University College London, Gower St, WC1E 6BT London, UK; 7 Imperial College London, South Kensington, SW7 2AZ London, UK

**Keywords:** Social accountability, Health inequalities, Health inequity, Medical schools, Medical curriculum, Medical education, Community engagement, Advocacy

## Abstract

The role of medical schools is in a process of change. The World Health Organization has declared that they can no longer be ivory towers whose primary focus is the production of specialist physicians and cutting edge laboratory research. They must also be socially accountable and direct their activities towards meeting the priority health concerns of the areas they serve. The agenda must be set in partnership with stakeholders including governments, health care organisations and the public.

The concept of social accountability has particular resonance for the Bar Ilan Faculty of Medicine in the Galilee, Israel’s newest medical school, which was established with a purpose of reducing health inequities in the Region. As a way of exploring and understanding the issues, discussions were held with international experts in the field who visited the Galilee. A symposium involving representatives from other medical schools in Israel was also held to extend the discourse. Deliberations that took place are reported here.

The meaning of social accountability was discussed, and how it could be achieved. Three forms of action were the principal foci – augmentation of the medical curriculum, direct action through community engagement and political advocacy. A platform was set for taking the social accountability agenda forward, with the hope that it will impact on health inequalities in Israel and contribute to discussions elsewhere.

## Introduction

In 2008 the World Health Organisation (WHO) published a report written by its Commission on Social Determinants of Health. Entitled *Closing the Gap in a Generation*[1]*,* it reported that many of the differences in health between and within countries stem from the social environment where people are born, live and age. The Commission recommended a two-pronged approach to redressing health inequities – improving people’s daily living conditions and tackling inequitable distribution of power, money and resources.

Although medical professionals can only have a limited role in implementing these recommendations, they nonetheless have a clear responsibility to address health inequities in the course of their work. This challenge was taken up by pioneering medical schools, many of whom were new schools. They were established with a paradigm shift - embedding improvement in community health with innovations in health professional education, with delivery and health policy at the core of their business. These schools engaged initially in establishing a network of community orientated medical schools, of which Ben Gurion University of the Negev was one, and evolved into an official relationship with the WHO as The NetWork [2].

The concept was taken further at a groundbreaking international meeting in South Africa in 2009 bringing together representatives of 130 organisations and individual experts from around the world with responsibility for health education, professional regulation and policy making to produce a Global Consensus for Social Accountability of Medical Schools (GCSA 2010) [3]. The Consensus underlined the social obligation for medical schools to direct their activities to the priority health concerns of the areas they serve, adapting to local context and priorities. The recognition and widespread uptake of this wider concept of Social Accountability and its centrality as a measure of underpinning “excellence” of an educational institution has been exemplified by recent development of awards in this category, with the Northern Ontario School of Medicine Canada and Southern Illinois University evidencing sustained global models of achievements [4,5].

*Closing the gap in a generation*[1] and the *Global Consensu*s [3] have special resonance for the Bar Ilan Faculty of Medicine in the Galilee, Israel’s newest medical school, which was established in 2011 with a vision to address the striking health inequalities in the Galilee Region. Local challenges involve a diverse population of Arab, Jewish and immigrant communities, and the distance from major tertiary care facilities. As part of a strategy to elucidate and map out a course of action we met with international leaders in the field to explore the issues and gain a greater understanding of how medical schools are taking up the challenge globally. A symposium was also held to promote a discourse on social accountability in Israeli medical schools more broadly and to exchange ideas and efforts [6].

This discussion document reflects some of the ideas and discussion that took place. Issues that were explored included: what is meant by social accountability; how it can be achieved; the role of medical education, direct action and advocacy; and how we can measure success over time.

### What is meant by social accountability?

The World Health Organisation defines social accountability as the obligation for medical schools to direct their activities towards meeting priority health concerns that are agreed in partnership with stakeholders nationally and locally. In many ways it is the next stage in the service, research and education revolution started several decades ago by the Community Oriented Primary Care movement [7], developed in conjunction with the Hebrew University, and builds on the approach taken in establishing the Ben Gurion medical school in the Negev [8]. However, despite these historical milestones, there was a challenge in agreeing what social accountability means in practice and considering how our efforts align with the WHO definition.

A recent article by Charles Boelen et al. [9] proved useful in helping differentiate between social responsibility, responsiveness and accountability. According to Boelen, medical schools can claim that they are *socially responsible* when they teach about health inequalities and the impact that social determinants have on health. *Social responsiveness* goes a step further as it involves taking action in response to societal or local community health needs. Abstracts submitted to the symposium (see Table 1) suggest that in Israel we are achieving a degree of social responsiveness at least, with eight abstracts describing educational activities that helped students acquire relevant skills, and twelve presenting programs run for the good of local communities. Encouragingly, abstracts came from departments of emergency and trauma medicine, surgery, psychiatry, psychology, student bodies, and not only medical education and public health.

**Table 1:** Abstracts submitted by Israeli medical schools to the symposium (Total number = 33)

Categorisation of the abstracts*		**Target population**	
Vision and values	7	General disadvantage	8
Medical school curriculum	8	Vulnerable children	6
Community engagement	12	Medical profession	6
Impact	3	Disabled or elderly	4
Other	7	Mentally or chronically ill	3
		Other	6

*some abstracts related to more than one category.

True social accountability, however, means more than this - it involves working in partnership with the population, both around their perceived health needs and to define the medical school’s academic agenda. This goal is still some way off for Israeli medical schools.

### How can social accountability be achieved?

Social accountability requires not only an ideology, but an ideology with evidence in the realm of the social determinants of health. There was general agreement that the ideology incorporates human rights, solidarity, pluralism, and empowerment. Equality in services, however, is not the desired end – proportionate universalism should be the goal, where services are augmented for those in greater need [1].

How medical schools can translate the ideology into effective action was much debated. According to TheNET [10], an international consortium of health professional institutions who are committed to achieving health equity [10] and AMEE, the Association of Medical Education in Europe [5] medical schools need an explicit stated vision and values that provide direction. Differing opinions were voiced regarding the need for the drive to come from the ‘top’. Some felt that lasting change and high-level partnership with key stakeholders can only occur when the medical school has an expressed strategy. Others offered a dissenting view supporting a ‘bottom-up’ approach [11] where the vision may have to grow from creativity and energy at the grass roots. Clearly a ‘bottom up’ approach may be the only way for medical schools which maintain a more narrow traditional biomedical focus.

Whether driven ‘top down’ or ‘bottom up’, there are three areas where a medical school can take action. The first is in educating their students to become capable and competent in grasping the physician’s role in tackling health inequities. The second is through direct engagement with local communities, and the last and perhaps more controversial is in the arena of political advocacy.

### Medical education

There is currently a mismatch between education and health system needs. The starting point must be what society wants and needs from its doctors – and this is more than being diagnosticians and prescribers of medicine. We need to ensure that students leave equipped with the relevant skills and attitudes to tackle health inequalities [12]. While there are any number of learning opportunities and experiences that we can provide, clearer definition of the specific competences is needed. These need to go beyond cultural competence and include leadership and advocacy. Social sensitivity and awareness are not enough; we need to develop doctors who can lead and innovate in health care. A paradigm shift is needed towards transformative education – where doctors are skilled to use their education to purpose and become agents of change. We need to develop doctors who can lead and innovate in health care and health promotion in its broadest sense.

Once there is clarity about the qualities that physicians of the future need, the curriculum can be designed. Tackling health inequalities is a major component, but theoretical knowledge has to be translated into how to ameliorate the impact of social determinants on patients’ health. This requires development of a faculty who are capable of teaching these skills. One way may be to identify ‘champions’ among doctors known to have an eye to the community. While family doctors often take a lead, allies may be found in specialties such as orthopaedics, emergency medicine and others where serious medical conditions caused or exacerbated by social determinants are often encountered. The challenge is the clinical years and there was agreement that a ‘golden thread’ running from preclinical courses to clinical clerkships is likely to be most effective. Case discussions that highlight health promotion, protection and prevention alongside diagnosis and treatment are one way to integrate concepts and management. The Clinico-epidemiological Conference [13], with the biopsychosocial approach at its core, is an example of how this can happen in practice.

There is an increasing acceptance that education and training around the social determinants of health and healthy equity should be an essential and universal component of health professional curricula and should be introduced early in training [12]. In order to develop leaders of the future enriched opportunities could also be more widely offered. Special electives or ‘pathways’ in social accountability could be a way of encouraging those students with interest and inclination to develop a career track in the area.

Lastly is the issue of ascertaining if competences are acquired. This is a demanding area, and a recent review indicates that while there is extensive literature on service learning as an educational approach, there is a need for more rigorous evaluation of the acquisition of competences [14].

Selection of our future doctors was an area that received attention too. In Israel, students are selected on academic merit, tempered with assessment of their values and sensitivity. Some medical schools outside of Israel are widening access by preferentially accepting students from underserved groups. Others are attempting to match doctors to health system needs by offering incentives for graduates to stay in underserved areas. Some discussion took place around whether the selection of students should be made involving society more in the decision making process.

### Direct action

Implicit in the WHO declaration is the requirement that medical schools must avoid being gated communities and ivory towers. They need to be involved with the communities where they are located. As medical schools are not traditionally prone to partnership, the concept of working with community organizations may be new, particularly for the higher echelons of medical school administration. The wider community needs to be involved in medical schools’ activities, through community representation on University committees and looking to the community for advice in curriculum planning.

There are different ways that medical schools can engage with their communities as exemplified by Northern Ontario medical school, Southern Illinois University and others [2,4,9].

Two were presented at the symposium, offering different approaches within Israel to partnership working. *Women for their Health*, a Hebrew University-Hadassah Hospital initiative, aims to improve the health of elderly women through professionals working with community organizations. Local needs are identified through focus groups and volunteers are trained to organize activities. In this way women’s leadership is fostered in addition to promoting women’s health.

In the Bar Ilan Faculty of Medicine in the Galilee, Project Raphael engages with the community by providing academic support and seedfunding to local organisations so that they can pilot innovative ideas to promote health. The projects are selected through a process that involves community leaders, using criteria of innovation, grassroots, sustainability, need and potential impact. Over time it is hoped that the needs of vulnerable populations will be addressed, and a network of community organisations created with the Faculty at the hub.

Another way that medical schools can engage is through student volunteerism, a process that benefits the community and the students themselves. One outstanding example is Project Lavi at the Hebrew University which was set up by a student in 2004. Linking medical students with patients hospitalized for long periods of time, it provides much needed support and personal contact for the needy. In the past year alone 75 students have volunteered in nine different departments. A further example of volunteerism is the Na’aseh v’nishma programme at Bar Ilan, a required course for students which couples community work with learning public health skills.

### Political advocacy

Political advocacy is the third arena for social accountability, and was felt to be the most controversial as it went beyond the traditional education and research remit of medical schools. Ideally advocacy is a dynamic two way process where the community becomes more involved with the university and the university more involved in the community – giving empowerment to both. Political advocacy receives less of our immediate attention, perhaps in part because health service development and distribution of resources are beyond our responsibilities, or beyond our expertise. Nonetheless medical schools have a role to play in drawing the attention of policy makers to health inequities. Activists and political lobbyists are needed, and doctors and medical schools form a potentially important and powerful lobby. An opportunity was provided during discussions around the establishment of the medical school in the Galilee when affiliated hospitals were asked about resources they needed to transform them into teaching hospitals. Facilities, equipment and specialized staff were requested but it could also have been an opportunity to address health inequalities more broadly. Interpreting services or health promoting activities might have been highlighted alongside the need for MRI scanners and more beds.

There is some debate regarding the stage at which advocacy skills and experience should be offered. Some argue that it is an important component of student education [15-17] and should be introduced at this formative stage of the doctor’s identity. Practically this is likely to be difficult given the heavy demands of the conventional curriculum, although discussion through case based learning can lay some foundation. Providing the experience during residency training may be more appropriate, although the opportunity is generally offered to those interested in developing a special interest rather than advocacy being a universal component of training [18].

### How can we ascertain progress?

Introducing the concept of social accountability into medical schools requires change in strategy, organizational culture and the curriculum. Ultimately one hopes that epidemiological health indicators will improve. However even when success is achieved, it will be hard to determine the extent to which medical schools are responsible. In the meantime we need to measure progress and results in the areas of medical education and community engagement, and determine the costs. This needs to be considered in the context of a wider health economic analysis, as examined by Northern Ontario medical school [19], which was established with an explicit intention to impact on the area where it is located.

Tools are needed to assess medical students’ attitudes and skills, their cultural competence and their career intentions both during training and following graduation. We need to track graduates over time to monitor their work with culturally diverse and disadvantaged populations, and their activity in promoting equity. At the symposium, a remarkable study was presented comparing the social medicine involvement of over 1400 graduates from across the country [20]. It demonstrated that Ben Gurion University of the Negev medical school, which was established with an emphasis on primary care, had indeed achieved higher rates of social involvement.

Involvement with local communities can also be mapped. At least one tool has been developed that can be used to study the impact of academic partnerships on organisations and the populations they serve [21]. The impact on health services also needs study, and both forms of evaluation will probably benefit from employing a mixed methodology involving both qualitative and quantitative research.

### Next steps

A number of messages emerged from our discussions. Firstly, there was assent that courage and creative energy are required to take the health equity agenda forward in medical schools. The agenda itself needs to be driven by ideology with evidence in order to move forward, and it seems that Israel is on the way to contributing significant evidence to the international literature. Lastly results cannot be achieved by academic enthusiasts alone; the public voice also needs to be heard to bring about effective and sustainable change.

The symposium helped us understand that the scope of activity in Israel was greater than we had previously believed. We would do well to see ourselves as a community, joining forces, and sharing and exchanging knowledge and tools so amplifying the impact.

Professor Murdoch Eaton introduced us to the Nguni Bantu concept of Ubuntu in which every citizen is responsible for promoting individual and social wellbeing, and it is society that gives human beings their humanity. We contributed our own heritage of solidarity - ‘kol Israel arevim ze la’ ze’ and ‘tiqqun olam’^a^ - to the discussion, and indeed perhaps those are good mottos on which to move forward and increase efforts towards contributing to a reduction in health inequities.

## Endnote

^a^Kol Israel arevim ze la’ ze’ - All Israel is responsible one to another - is a Talmudic idiom (Sifra Behukotai 7:8) originally implying shared responsibility for proper social and moral conduct in the community, but used in more recent centuries to connote mutual responsibility for social welfare.

“Tiqqun ’olam” was translated in the title of Jonathan Sacks’ book as “To heal a fractured world – the ethics of responsibility” (Schocken, New York, 2005).

## Competing interests

None of the authors have declared competing interests.

## Authors’ contributions

All the authors made substantial contributions to the discussions underpinning this paper. The manuscript was drafted by MCJR and the remaining authors contributed critical revisions and gave final approval of the version submitted to IJHPR. They all agree to be accountable for all aspects of the work and in ensuring that questions related to the accuracy or integrity of any part are appropriately investigated and resolved. All authors read and approved the final manuscript.

## Authors’ information

Professor Mary Rudolf is the lead for Public Health at the Bar Ilan University Faculty of Medicine in the Galilee since 2012, and visiting Professor of Child Health at the University of Leeds, UK. Areas of special interest include childhood obesity, paediatric education and social accountability of medical schools. Professor Shmuel Reis is a Family Physician in a rural health center, head of the Faculty Development Unit and the Clinical Skills Course director of the Bar Ilan University Faculty of Medicine in the Galilee. Professor Trevor Gibbs is an Independent and WHO Consultant in Medical Education, Primary Care and Adolescent Health. He also holds the position of Development officer for AMEE. His main areas of interest are in International medical curricula, Competency-Based Education, Social Accountability and Nutrition and Health. Professor Deborah Murdoch-Eaton is Dean of Medical Education, at The Medical School, University of Sheffield, UK. Her academic interests focus on Global Health, developing students' potential and individuality, embedding Social Accountability within medical education, and role of feedback in the development of learning skills. Professor David Stone is Emeritus Professor of Paediatric Epidemiology at the University of Glasgow, UK. Previous positions included Senior Lecturer in Epidemiology at Ben Gurion University of the Negev, Israel and Senior Medical Advisor to the Scottish Government’s Chief Medical Officer. Dr Michael Grady is an expert adviser to WHO and special adviser to the UK House of Commons Community and Local Government Select Committee. He advises Local authorities and municipalities nationally and internationally on implementation on action on the social determinants of health. Dr Anita Berlin is an inner London General Practitioner and a senior lecturer in primary care at University College London. She is curriculum lead for the new Social Determinants of Health Module and for Patient and Public Participation. Prof Mitch Blair is Consultant Paediatrician and Reader in Child Public Health Imperial College London and Northwick Park Hospital. He is undergraduate paediatrics course lead for Imperial medical students and has a strong interest in teaching Child Rights and Health Inequalities. Dr Jumanah Essa-Hadad has a PhD in Public Health from the University of Haifa. Research interests include web-based health education and health of minority groups and disadvantaged populations. Her background includes working with NGO's and civil society organizations at the grassroots level to promote public health programs. Ms Sivan Shohat holds a Master's degree in Sociology specializing in health care policy and the role healthcare organizations have in reducing health and healthcare disparities. Professor Michael Weingarten is Associate Dean for Medical Education at the Bar Ilan University Faculty of Medicine in the Galilee. A family practitioner by background, he has a special interest in medical ethics.

## References

[B1] CSDH Closing the Gap in a Generation. Health equity through action on the social determinants of health. Final report of the Commission on the Social Determinants of Health 2008 Geneva: World Health Organisation http://whqlibdoc.who.int/publications/2008/9789241563703_eng.pdf?ua=1

[B2] The NetWork http://www.the-networktufh.org (accessed 9 May 2014)

[B3] Global Consensus for Social Accountability of Medical Schools Dec 2010 http://healthsocialaccountability.org/ (accessed 8 May 2014)

[B4] Boelen B Woollard R Social accountability: the extra leap to excellence for educational institutions Med Teach 2011 31 614 619 21774646 10.3109/0142159X.2011.590248

[B5] Association for Medical Education in Europe ASPIRE awards http://www.amee.org/awards-prizes#aspiretoexcellence-award (accessed 12 May 2014)

[B6] Social accountability of medical schools: a symposium held at the Bar Ilan Faculty of Medicine in the Galilee. November 2013 Programme and abstracts accessible at: http://medweb.md.biu.ac.il/research/mary-rudolf/index.php/conferences

[B7] Nutting P Community Oriented Primary Care: from principles to practice 1990 Univ New Mexico Press

[B8] Porter B Seidelman WE The politics of reform in medical education and health services: The Negev Project 1992 New York: Springer 10.1097/00001888-199208000-00005 1497778

[B9] Boelen C Dharamsi S Gibbs T The social accountability of medical schools and its indicators Educ Health 2012 25 180 94 10.4103/1357-6283.109785 23823638

[B10] The Training for Health Equity Network. THEnet’s Social Accountability Evaluation Framework Version 1. Monograph I 2011 1 The Training for Health Equity Network http://thenetcommunity.org/category/reports/

[B11] Crisp BR Swerissen H Duckett SJ Four approaches to capacity building in health: consequences for measurement and accountability Health Promot Int 2000 15 99 107 10.1093/heapro/15.2.99

[B12] Institute of Health Equity Working for health equity: The role of health professionals 2013 https://www.instituteofhealthequity.org/projects/working-for-health-equity-the-role-of-health-professionals

[B13] Stone DH The clinico-epidemiological conference: a proposed new pedagogic tool for the integration of epidemiology and clinical practice J Public Health 2013 doi: 10.1093/pubmed/fdt094 10.1093/pubmed/fdt094 24006355

[B14] Mc Menamin R McGrath M Cantillon P MacFarlane A Training socially responsive health care graduates: Is service learning an effective educational approach? Med Teach 2014 36 4 291 307 doi: 10.3109/0142159X.2013.873118 10.3109/0142159X.2013.873118 24650270

[B15] Belkowitz J Sanders LM Zhang C Agarwal G Lichtstein D Mechaber AJ Chung EK Teaching health advocacy to medical students: a comparison study J Public Health Manag Pract 2013 [Epub ahead of print] PMID:24322841 10.1097/PHH.0000000000000031 24322841

[B16] Blair M Koury S De Witt T Cundall D Teaching and training in community child health: learning from global experience Arch Dis Child Ed Pract 2009 94 123 128 doi: 10.1136/adc.2008.142323 10.1136/adc.2008.142323 19654404

[B17] Blair M Training and education as a means of increasing equity in child health teaching of undergraduates Pediatrics 2003 112 747 748 12949341

[B18] Rudolf MCJ Bundle A Damman D Garner M Kaur V Khan M Robinson G Ruge S Waterston T Exploring the scope for advocacy by paediatricians Arch Dis Child 1999 81 6 515 518 10.1136/adc.81.6.515 10569972 PMC1718159

[B19] Exploring the Socio Economic Impact of the Northern Ontario School of Medicine Final Report – November, 2009 Centre for Rural and Northern Health Research Lakehead and Laurentian Universitieshttp://www.cranhr.ca/. (last accessed 8 May 2014)

[B20] Dopelt K Yahav Z Urkin J Bachner Y Davidovitch N The social role of the faculties of medicine: physicians’ perception of the dominant orientation of their medical studies and social involvement Harefuah 2014 153 2 87 91 24716425

[B21] King G Servais M Kertoy M Specht J Currie M Rosenbaum P Law M Forchuk C Chalmers H Willoughby T A measure of community members’ perceptions of the impacts of research partnerships in health and social services Eval Program Plann 2009 32 3 289 99 10.1016/j.evalprogplan.2009.02.002 19304326

